# Changes over time in the relationship between weight, body fat, motivation, impulsivity and eating behaviour

**DOI:** 10.1186/s12889-021-11402-7

**Published:** 2021-07-08

**Authors:** Paula Foscarini-Craggs, Rob Lowe, Michelle Lee

**Affiliations:** 1 Centre for Trials Research, Cardiff University, Cardiff, UK; 2 Psychology Department, Swansea University, Swansea, UK

**Keywords:** ‘Freshman 15’, Self-determination theory, Impulsivity, Weight change, Eating behaviour

## Abstract

**Background:**

University students are at a greater risk of gaining weight compared to others. We explored associations between changes in weight and a set of dispositional constructs related to eating behaviour: motivation, impulsivity, social comparison, and eating styles. We predicted that increases in controlled motivation, impulsivity, uncontrolled eating, emotional eating, and physical appearance comparison would be related to increased weight and body fat.

**Methods:**

First year students at a British university completed baseline (*n* = 196) and three-month (*n* = 163) measures of impulsivity, physical appearance comparison, motivation for following a healthy diet, eating styles, weight and body fat. Baseline-follow-up changes in these constructs were computed and subjected to cluster analysis.

**Results:**

Four participant groups were identified according to similarities in the way these constructs evolved over time. The *Losing* and *Gaining* groups tended to show opposing changes in key variables (physical appearance comparison, uncontrolled eating, motivation, weight, and percentage of body fat). Interestingly, two groups showed no change in weight and body fat but evidenced unique changes in key variables, indicating that individuals can have different psychological profiles but still maintain their weight.

**Conclusions:**

The study highlighted how stable weight maintenance arises from sets of interdependent constructs rather than variables in isolation, as well as emphasizing a need to take a person-centred approach to examining those at risk of weight gain and in developing interventions.

## Background

Moving from home to university represents a life change, often necessitating redevelopment of health-related habits. Environment changes can disrupt established habits because pre-existing behavioural cues are no longer present [1–3]. Attitudes, motivation, and poor self-control influence the development of unhealthy habits when in new environments [3–5]. This study examined changes in motivation, impulsivity, social comparison, and eating styles to identify patterns of change associated with weight gain in first-year university students.

Weight change in first year university students ranges from 0.83 kg to 4.5 kg [6, 7], and is independent of changed height [8–10]. University students gain weight quicker than their peers not in university [11]. Implicated in this weight gain are high-fat foods present in dining halls, frequent snacking, drinking alcohol [12–16] and a decrease in physical activity [7, 8, 17, 18]. However, not all students gain weight, suggesting specific risk factors are present.

Research has examined individual variables facilitating weight gain [6, 9, 17, 19], by exploring trends across the whole sample in specific eating traits (eating restraint, uncontrolled eating), and interpersonal relationships and dispositional factors (impulsivity and motivation) on weight gain. Studies looking at healthy habits in adults show that individuals with a given healthy habit tend to have other healthy habits [20, 21]. This suggests ‘sets’ or clusters of traits which support or maintain these healthy habits. The current study built on this research by examining a wider set of interrelated dispositional variables: motivation, impulsivity, eating styles, and social comparison. This study aimed to explore the extent to which these factors evolved over a 3-month period and combine together in their association with changing weight among new university attendees.

Regarding motivation, Self-Determination Theory [22] classifies the quality of motivation according to different orientations. Firstly, *controlling* which includes *external regulation* (motivated by reward or avoiding punishment) and *introjected regulation* (motivated by ego enhancement or avoiding guilt); secondly, *autonomous* which includes *identified regulation* (motivated by a goal) and *intrinsic motivation* (enjoyment of the behaviour) [22–24]. Autonomous motivation facilitates the development of healthy habits, with links to weight loss in students because it is goal oriented and driven by enjoyment of the behaviour [25–28].

Impulsivity can influence a person’s food choices through impairments in their executive function, such as an inability to defer gratification and increased disinhibition [27–32]. In students, impulsivity is related to higher consumption of saturated fats and snacking through uncontrolled and emotional eating [6, 33–36]. Negative effects of impulsivity can be mitigated by boosting executive function through increasing autonomous motivation [37–39].

Restrained eating is restricting dietary intake to lose or maintain weight [6, 19, 40]. However, there is no clear relationship between restrained eating and weight change in university students [10, 19, 41, 42]. For example, elevated dietary restraint is associated with weight loss [41] as well as weight gain [19]. The current study aims to determine whether other factors, operating in conjunction with restrained eating, place individuals at greater or lower risk of gaining weight.

Social comparison orientation is a tendency to compare and modify behaviour based on perceived norms [43–48] and is related to controlled motivation due to the pressure of normative influence [49–52]. Social comparison is associated with changed eating behaviour based on perceived physical appearance and body size norms of the reference group [53–56]. Thus, weight gain in students may be associated with social comparison and social activities centred around food.

Changes in psychological variables associated with lifestyle behaviours would be expected to lead to changes in those behaviours. For example, elevated impulsivity is associated with increased alcohol consumption in young adults [57, 58]. Changes in social groups and environment may also impact motivation [22, 24, 49]. Cognitive restraint and social comparison orientation may be influenced by students’ new social milieu. Students change their eating behaviour, and by extension dieting behaviour, based on friendship groups and this can further influence changes in physical appearance comparison [53, 59–61].

To summarise, entering university represents a critical life period, where young adults are at risk of developing unhealthy habits [60, 62, 63]. Our study builds on previous work by taking advantage of this critical period and longitudinally tracking *changes* over 3 months in both psychological traits and body fat/weight to identify *clusters* (sets) of psychological factors predisposing individuals to weight change. We hypothesized that new university students who increase body fat and/or weight will evidence decreased autonomous motivation (identified regulation, and intrinsic motivation) and decreased physical activity, but increased controlled motivation (external regulation and introjected regulation), impulsivity, physical appearance comparison, emotional eating, and uncontrolled eating. Those who decreased body fat and/or weight would show the opposite pattern.

## Method

### Participants

The baseline sample comprised 196 first-year students attending a UK university. There were three recruitment waves: Cohort A (*n* = 76), Cohort B (*n* = 82) and Cohort C (*n* = 38). For each, follow-up occurred 3 months later, resulting in a follow-up sample of *n* = 163 (83.2% retention). Participants were primarily recruited through the psychology department but university-wide emails were also used. Participants were eligible if they were enrolled in their first year of their first undergraduate degree. Every effort was made to ensure the study recruited participants with a range of body sizes. Psychology participants (89.28% of the sample) received study credits for each study session attended, whilst non-psychology participants received an entry to a draw for a cash prize for each attended session. Most participants who did not attend the second session did not respond to follow up reminders and only one participant declined to continue in the study. To address limitations in sample size, three cohorts were recruited. Ethical approval was given by the psychology departmental research ethics committee.

### Measures^1^

*Motivation for Eating* was assessed with a questionnaire measure of extrinsic and intrinsic motivation towards eating a healthy diet. It was a modification of the Self-Regulation for Exercise scale [64], done by replacing references to exercise with references to healthy diet. Subscales measure the four types of motivation from self-determination theory: external regulation (four items), introjected regulation (three items), identified regulation (four items) and intrinsic motivation (four items) [49]. Response options ranged from 1 (not true for me) to 5 (true for me) and scored by averaging responses for each subscale. Higher scores indicate elevated motivational style. Alpha coefficient can be found in Table 1.

**Table 1:** Alpha Coefficients for Study Variables at Each Time Point

Variable	T1	T2
Controlled Motivation		
External	0.84	0.88
Introjected	0.89	0.93
Autonomous Motivation		
Identified	0.73	0.72
Intrinsic	0.88	0.91
Impulsivity	0.84	0.87
Eating Styles		
Emotional Eating	0.83	0.85
Uncontrolled Eating	0.71	0.76
Cognitive Restraint	0.61	0.79
PACS	0.63	0.74

*Impulsivity* was assessed with the Barratt Impulsiveness Scale-11 [65], comprising 30 items with a four-point Likert scale ranging from 1 (rarely/never) to 4 (almost always/always). Items implying lower impulsivity are reverse-scored and then all items summed. Higher scores indicate greater impulsivity. Alpha coefficient can be found in Table 1.

*Eating Style:* cognitive and behavioural components of eating were assessed using the brief version of the Three-Factor Eating Questionnaire [40] which has 18 items. Responses are recorded using a Likert scale, with labels varying according to sub-scale. There are three subscales: emotional eating (a tendency to eat to cope with negative emotions, three items), uncontrolled eating (an inability to effectively regulate food intake, nine items), and cognitive restraint (limiting food intake to control weight, six items). Subscales scores are derived by reverse coding items indicating less of the particular eating style and then summing across all items. Higher scores indicate a greater tendency to exhibit that particular eating style. Alpha coefficient can be found in Table 1.

*Physical Appearance Comparison* was assessed using The Physical Appearance Comparisons Scale (PACS) [66]. This measures the respondent’s tendency to compare their physical appearance to others within their social circle and across different social situations. The questionnaire contains five items and uses a five pointe Likert scale ranging from 1 (never) to 5 (always). Items indicating a tendency to not compare are reversed coded and then all items are summed. Higher scores indicate a greater tendency to compare physical appearance with others. Alpha coefficient can be found in Table 1.

*Time spent doing Physical Activity* was assessed using a modified version of the 7 Day Physical Activity (PA) Recall [67], which measures level of physical activity over the preceding week. The original version comprises a one-to-one interview wherein participants recall how many days of the week they were physically active, for how long, and how strenuously. A modified version was used to enable self-reporting and was based on the method used by Lowe, Eves, and Carroll [68]. This comprised a list of common physical activities; participants indicated how many hours/minutes they pursued each activity for each day of the preceding week. In the current study, the measure assessed *commitment* to physical activity. Given the study’s focus on motivation, there was a potential confound between time spent on an activity and energy expenditure, either of which can represent commitment, but which may be obscured by combining them in the normal way. We used time spent in activity to index commitment based on the assumption that someone committed to an activity will spend as much time as possible in its pursuance. To derive a single score, time (in minutes) was summed across all activity bouts.

*Anthropometric measurements and demographic data*: height, weight, and percentage of body fat were measured at each time point. Height (meters) was measured on a stadiometre (SECA laboratory Scales). A set of Tanita BF-350 scales [69] measured weight (kilograms) and percentage body fat, the latter indexed via bioelectrical impedance [69]. Age and gender were recorded during the baseline session.

#### Procedure

Participants were initially recruited over a month at the start of the academic year. Participants were contacted to attend the follow up session in the middle of January, following the Christmas break and exam period, via email and text message. Participants who did not respond were emailed or texted weekly across the following month to encourage them to attend a follow up session. At baseline and time-2, participants completed questionnaires on SurveyMonkey prior to being weighed. To obtain an accurate weight and for the use of the bio-impedance, participants were asked to remove their shoes, socks, jacket or jumper, and to take anything heavy (such as mobile phone or keys) out of their pockets prior to stepping on the scale.

### Statistical analysis

The focus of the study was to identify unique clusters of people based on their degree of weight/body fat change and change in related key psychological variables during the first 3 months at university.

Following recommendations of Tabachnick and Fidell (2007), missing data that was limited to a single skipped item was replaced using the Expectation Maximization method and then scale values were computed for that questionnaire. At baseline and time-2, 8.23 and 11.28% respectively of participants had one item replaced. Analyses were run with and without missing data and central findings were overall the same. Therefore, the set with the replaced data was kept so as to maximise sample size. At time-1, impulsivity (3 participants), physical activity (4 participants), and weight (3 participants) had data points omitted due to either missing data, or the data point was classified as an outlier (±3SD). At time-2, impulsivity (2 participants), physical appearance comparison (1 participant), physical activity (1 participant), weight (7 participants), and percentage of body fat (5 participants) had data points omitted due to missing data or being outliers. Because we were looking at changes in clusters of variables, only participants with complete datasets were entered into the cluster analysis. This resulted in a total sample of 142 participants included in the final analysis.

The aim of this study was to explore how sets of traits change together over time and whether these changes are associated with changes in weight and body fat. The change in each study variable was computed as the unstandardized residual between its baseline and time-2 score. The residual scores were then used in a cluster analysis to identify groups of participants who had a similar profile of change over time. Following Clatworthy et al. [70], a three step cluster analysis was used. First, a Ward analysis (squared Euclidian distances with no specified number of clusters) identified 4 clusters via the dendrogram and cluster coefficients. A second Ward analysis, specifying four clusters, determined cluster centroids for seeding the final K-means analysis used to identify final group membership for the (four) clusters.

The mean of each study variable in a given cluster was compared to the mean across the remaining three clusters. Differences were indexed by their effect sizes (Cohen’s *d*). Salient variables had a medium to large effect size (Cohen’s *d* = +/− .05; equivalent to a significance level of *p* ≤ .01). In this way, it was possible to identify which variables were especially (un) characteristic as the cluster evolved over time. Effect sizes were used to address potential biases due to the sample size.

## Results

Means and standard deviations for critical study variables at baseline and follow up, along with age and gender, are shown in Table 2.

**Table 2:** Means (SD) at Time 1 and Time 2 for total sample

	T1	T2	Change
Controlled Motivation			
External	1.86 (±0.84)	2.10 (±0.94)	0.23(±0.87)
Introjected	3.00 (±1.29)	3.03 (±1.28)	−0.01(±1.08)
Autonomous Motivation			
Identified	3.80 (±0.91)	3.77(±0.83)	−0.04(±0.71)
Intrinsic	3.31 (±1.01)	3.42 (±1.09)	0.08(±0.82)
			14.26
Impulsivity	63.61(±10.16)	78.14(±14.90)	14.26(±10.61)
Eating Styles			
Emotional Eating	7.00(±2.77)	6.86(±2.64)	−0.13(±2.32)
Uncontrolled Eating	20.99(±5.79)	21.31(±5.73)	0.40(±4.13)
Cognitive Restraint	14.61(±4.11)	15.09(±4.75)	0.35(±3.83)
PACS	15.55(±3.93)	15.17(±4.11)	−0.33(±2.78)
PA (min/week)	490.39(±344.25)	458.32(±367.10)	−26.07(±311.28)
Weight (Kg)	64.36 (±11.68)	62.96 (±11.04)	0.41 (2.44)
Body Fat (%)	23.24 (±9.23)	23.92(±8.99)	0.70(±2.92)
BMI	23.20 (± 3.81)	23.47 (±4.58)	0.03(±0.90)
Height (m)	1.67(±0.08)	1.67(±0.8)	–
Age	19.04 (±2.23)	–	–
Gender	151 (77.04%) Females	120 (76.92%) Female	–

### The gaining cluster

**Fig. 1:**
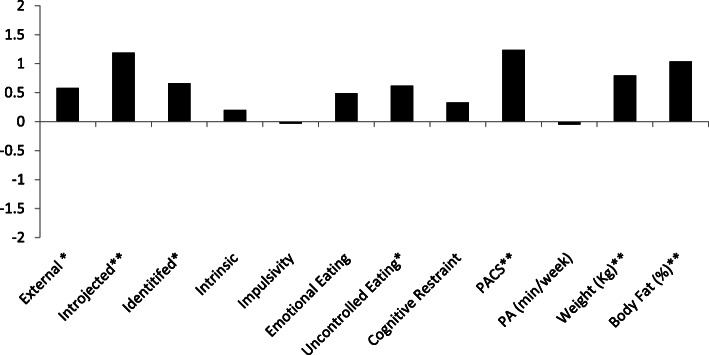
Cohen’s *d* values for the Gaining Cluster. *Represent a medium effect size (*d* = ±0.5). **Represents a large effect size (*d* = ±0.8)

Participants (*n* = 48) in this cluster evidenced a mean increase of 1.37 kg and 1.28% body fat. Across the cluster, there were increases in physical appearance comparison, external regulation, introjected regulation, identified regulation, as well as uncontrolled eating (See Fig. 1 for effect size values and Table 3 for means and change values).

**Table 3:** Means (SD) and change score of the Gaining Cluster, and The Losing Cluster

	Gaining Cluster			Losing Cluster		
	T1	T2	Change	T1	T2	Change
Controlled Motivation						
External	1.88 (±0.94)	2.22 (±0.98)	1.47^a^	2.15 (±0.82)	2.47 (±0.80)	0.32
Introjected	2.94 (±1.31)	3.41 (±1.10)	0.46^a^	3.64 (±1.05)	4.20 (±1.25)	0.29
Autonomous Motivation						
Identified	3.65 (±1.04)	3.68 (±0.82)	0.02^a^	4.13 (±0.67)	4.20 (±0.60)	0.07
Intrinsic	3.04 (±1.07)	3.25 (±1.13)	0.22	3.55 (±0.75)	4.12 (±0.66)	0.57^a^
Impulsivity	63.71 (±9.98)	78.18 (±14.95)	14.47	64.40 (±11.84)	78.93 (±15.00)	14.53
Eating Styles						
Emotional Eating	7.57 (±2.78)	8.43 (±2.19)	0.86	7.80 (±3.14)	8.07 (±2.69)	0.27
Uncontrolled Eating	21.25 (±5.86)	23.24 (±5.78)	1.98^a^	24.07 (±6.25)	22.53 (±6.51)	−1.53^a^
Cognitive Restraint	13.82 (±4.39)	14.92 (±4.06)	1.10	15.60 (±3.83)	20.93 (±3.61)	5.33^a^
PACS	15.43 (±4.32)	16.90 (±3.93)	1.47 ^a^	17.20 (±4.92)	15.87 (±4.60)	− 1.33^a^
PA (min/week)	494.61 (±355.33)	449.22 (±339.93)	− 45.39	357.13 (±239.99)	295.00 (±246.72)	−62.13
Weight (Kg)	62.95 (±10.13)	64.32 (±10.46)	1.37^a^	68.80 (±13.25)	65.35 (±12.82)	−3.45^a^
Body Fat (%)	25.50 (±9.27)	26.79 (±9.27)	1.28^a^	29.06 (±7.49)	27.45 (±8.09)	−1.61^a^

^a^ represents meaningful changes

### The losing cluster

**Fig. 2:**
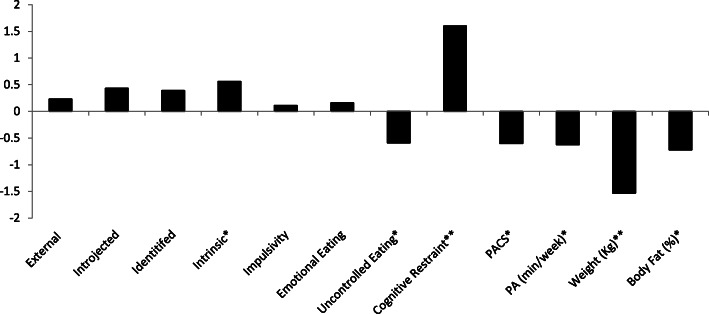
Cohen’s *d* values for the Losing Cluster. *Represent a medium effect size (*d* = ±0.5). **Represents a large effect size (*d* = ±0.8)

Participants (*n* = 25) in this cluster evidenced a decrease of 3.45 kg, and 1.61% of body fat. This cluster also showed decreases in physical appearance comparison, uncontrolled eating, and the amount of time spent doing physical activity. There were increases in intrinsic motivation and in cognitive restraint (see Fig. 2 for effect size values and Table 3 for means and change values).

### The no change-high autonomous cluster

**Fig. 3:**
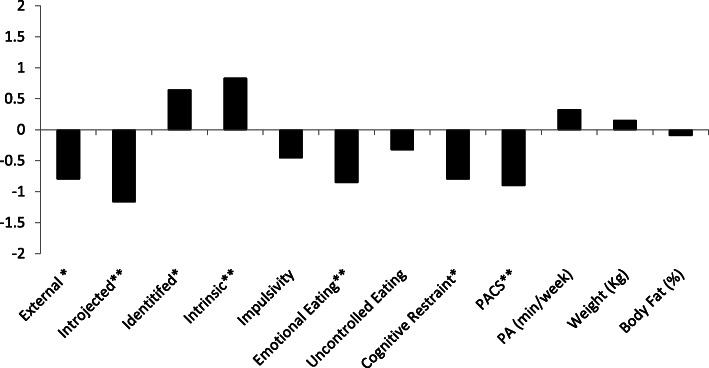
Cohen’s *d* values for the No Change-High Autonomous Cluster. *Represent a medium effect size (*d* = ±0.5). **Represents a large effect size (*d* = ±0.8)

Participants (*n* = 34) in this cluster showed a modest increase of 0.46 kg and 0.93% of weight and body fat but it was not statistically meaningful. Across the cluster, there were decreases of in physical appearance comparison, external regulation, introjected regulation, emotional eating, and cognitive restraint. There were increases in identified regulation, and intrinsic motivation (see Fig. 3 for effect size values, and Table 4 for means and change values).

**Table 4:** Means (SD) and change score of the No-Change and High Autonomous Cluster, and The No Change-Low Autonomous Cluster

	No Change-High Autonomous			No Change-Low Autonomous		
	T1	T2	Change	T1	T2	Change
Controlled Motivation						
External	1.78 (±0.80)	1.87 (±0.84)	0.08^a^	1.80 (±0.78)	2.10 (±1.06)	0.30
Introjected	2.93 (±1.29)	2.69 (±1.22)	−0.25^a^	2.88 (±1.37)	2.42 (±1.30)	−0.46^a^
Autonomous Motivation						
Identified	3.87 (±0.78)	4.02 (±0.68)	0.16^a^	3.78 (±0.94)	3.24 (±0.95)	−0.54^a^
Intrinsic	3.41 (±1.01)	3.80 (±0.93)	0.39^a^	3.52 (±0.96)	2.63 (±0.98)	−0.88^a^
Impulsivity	62.60 (±8.93)	77.14 (±12.99)	14.54	64.92 (±11.98)	79.54 (±18.56)	14.62
Eating Styles						
Emotional Eating	6.30 (±2.67)	5.26 (±2.33)	−1.04^a^	6.78 (±2.49)	6.15 (±1.91)	−0.65
Uncontrolled Eating	19.58 (±5.48)	19.56 (±5.39)	−0.02	21.38 (±5.46)	20.19 (±4.66)	−1.19
Cognitive Restraint	14.66 (±3.74)	14.78 (±4.79)	0.12^a^	15.46 (±4.31)	12.65 (±3.91)	−2.81^a^
PACS	14.94 (±3.39)	13.90 (±3.63)	−1.04^a^	16.00 (±3.36)	13.81 (±3.90)	−2.19
PA (min/week)	551.06 (±383.43)	529.24 (±458.09)	−21.82)	442.31 (±273.85)	434.04 (±238.18)	−8.27
Weight (Kg)	63.16 (±10.83)	63.62 (±11.39)	0.46	64.33 (±12.34)	64.79 (±13.07)	0.46
Body Fat (%)	18.98 (±8.70)	19.91 (±7.97)	0.93	23.63 (±7.86)	23.97 (±8.12)	0.34

^a^ Represents meaningful changes

### The no change-low autonomous cluster

**Fig. 4:**
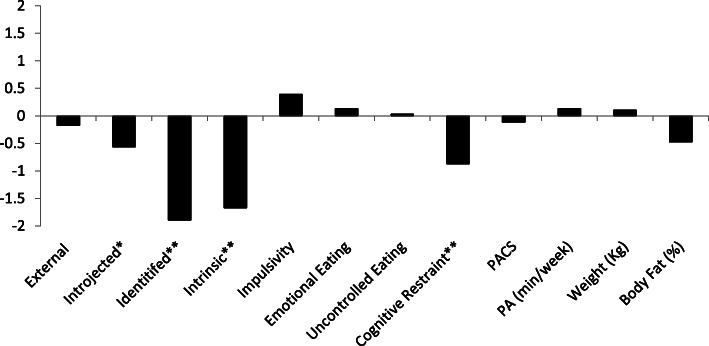
Cohen’s *d* values for the No Change-Low Autonomous Cluster. *Represent a medium effect size (*d* = ±0.5). **Represents a large effect size (*d* = ±0.8)

For participants (*n* = 35), the increase of 0.46 kg and the decrease of 0.34% of body was not statistically meaningful. Across the cluster, respondents evidenced decreased introjected regulation, identified regulation, intrinsic motivation, and cognitive restraint (see Fig. 4 for effect size values, and Table 4 for means and change values).

## Discussion

The aim of current study was to explore how a set of dispositional factors changed and how these changes related to weight and body fat change during the first 3 months of university. Studies using regression-based analysis of the whole sample may highlight findings that generalize across the sample but may miss relationships important for sub-groups due to individual variation. By contrast, our study aimed to identify variables important for sample sub-groups – the evolution over time of predictor and outcome variables that were especially salient to each sub-group. This would allow for a more nuanced understanding of how groups of people may differ in the set of variables relevant to their weight change. Furthermore, by looking at how predictor variables clustered in terms of their relevance to specific participant sub-groups, our analysis acknowledges that critical study variables may be co-dependent - operating as non-independent sets rather than in isolation.

It was predicted that people who gained weight and body fat would be typified by increased controlled motivation (external regulation and introjected regulation), decreased autonomous motivation (identified regulation and intrinsic motivation), increased impulsivity, increased emotional eating, increased uncontrolled eating, increased physical appearance comparison, and decreased physical activity. By contrast, those who lost weight and body fat would be typified by a converse pattern. There was partial support for the hypotheses; two clusters evidenced changes in weight/body fat with several variables following the predicted patterns. It should be noted that, overall, participants in the current study are slightly lighter than population averages [71] but are comparable to previous research [6, 35] looking at weight change in university students in the UK.

The *Gaining cluster* was characterized by increases in weight/body fat across the study period. The weight gain was approximately double what previous UK research has reported over the same timeframe (0.83 kg [6];) but less compared to North American studies [7, 72]. This cluster also gained approximately double the amount of body fat compared to what was reported by Hoffman et al. [5] (0.9%), one of only a few studies we are aware of that reported change in body fat among university students.

The *Gaining cluster* evidenced an increase in identified regulation. Despite evidence showing that identified regulation is related to weight loss and greater behavioural persistence [73, 74], the benefits may have been offset by the increase in controlled motivation (which was predicted to be associated with weight gain). Furthermore, the negative effects of controlled motivation were compounded by an increase in physical appearance comparison. Students high in physical appearance comparison are more likely to focus on external cues, such as perceived norms, and are more likely to feel pressure to act in accordance to those norms (i.e. controlled motivation) [24, 44, 46, 47, 52, 75]. In the context of the current study, there would be a decrease in healthy eating when no longer in a social group that encourages it. A further risk factor for the *Gaining cluster* was the increase in uncontrolled eating. Findings were consistent with our prediction and accord with previous research [6, 33].

Regarding the *Losing cluster*, a loss of 3.45 kg and of 1.61% body fat were key differentiating characteristics compared to the other clusters. Previous research reported similar losses with both Serlachius et al. [35] and Jung et al. [76] reporting weight loss of between 2 and 3 kg. The *Losing cluster* showed an increase in intrinsic motivation – a factor identified as important for following a healthy lifestyle [25–27]. Along with increased intrinsic motivation, the *Losing cluster* also showed a notable reduction in physical appearance comparison, supporting the study hypotheses. We speculate that individuals in the *Losing cluster* were more likely to follow a healthy diet because they chose to do so and were less influenced by their social groupings and accompanying perceived norms [13, 49, 52]. Future research may want to explore this further by looking at the way those who are intrinsically motivated to eat healthily make food decisions while eating in social groups.

Compared to other clusters, the *Losing cluster* also showed a notable increase in cognitive restraint and a decrease in uncontrolled eating. Pliner and Saunders [19] found that cognitive restraint was associated with weight gain. In contrast, Delinsky and Wilson [41] found that a combination of cognitive restraint and concern for weight gain was positively related to weight loss in first year university students. Our interpretation of the current findings is that cognitive restraint acted as protection against weight gain because the students were less likely to binge eat (shown by a decrease in uncontrolled eating) and were more aware of and enjoyed a healthy diet (seen by the increase in intrinsic motivation). This highlights the need to study related sets of psychological factors when exploring weight change; health-related behaviours may arise from interacting psychological factors rather than factors in isolation.

Finally, the *Losing cluster* also showed a decrease in the amount of time spent doing physical activity. This contradicts previous research in university students [17, 18, 76] but is more line with recent research highlighting how physical activity is not an effective method for weight loss because food intake increases to balance the energy deficit arising from increased physical activity, and increases in muscle mass can offset any weight loss through fat reduction [77–81].

Findings also identified two clusters where weight/body fat remained largely stable: A *No change-Low autonomous motivation cluster* and a *No change-High autonomous cluster*. The *No change-Low autonomous motivation cluster* showed a non-meaningful gain of half a kilogram and a negligible change in body fat. This cluster showed decreases in introjected regulation, autonomous motivation, and cognitive restraint. Even though it would have been expected that a decrease in autonomous motivation would be related to an increase in weight, those in this cluster may have been protected because there was an accompanying decrease in introjected regulation, a facet of controlled motivation [73, 74, 82–84]. That is to say, it appeared that participants were not motivated to eat healthily but it is also likely they were not eating unhealthily either. They were also less at risk of binge eating through the decrease in cognitive restraint [19, 41, 85, 86]. The *No change-high autonomous cluster* showed a small non-meaningful gain of half a kilogram and just over half a percent of body fat. There was a decrease in controlled motivation which was accompanied by a corresponding increase in autonomous motivation and a decrease in physical appearance comparison. This meant they were less likely to focus on external cues. People in this cluster also evidenced a decrease in cognitive restraint and emotional eating. Once again, the risk of weight gain associated with cognitive restraint may have depended on changes in other traits. In this case, even though the *No change-high autonomous cluster* was not restricting diet to control weight, they were less likely to turn to food during times of emotional distress (indicated by the decrease in emotional eating) and they were autonomously choosing to eat healthily. Again, findings are consistent with health behaviours arising from sets of interacting psychological factors rather than factors in isolation.

Consistent with previous research, the current findings showed that entering university represents a critical period where healthy habits are in flux. The results indicated that when individuals are in a new environment, psychological traits can change and may influence the development of healthy habits. When developing interventions, it is important to look at a collective of psychological variables because, as the current results indicate, sets of variables appear to operate together and interactions could impact on whether a variable is considered a risk factor for weight gain. An interaction between psychological variables may also help explain seemingly contradictory research findings. Furthermore, the results of the current study reinforce the need to take a person-centred approach and not assume that groups of individuals are homogenous in the way that underlying psychological variables change despite similar outcomes. In the current study there were two groups that were able to maintain their weight but had different underlying psychological traits.

Notwithstanding the above, the study had several limitations. First, we examined only the first 3 months at university, which limits conclusions regarding longer-term risks of weight/body fat gain. Even though previous research has indicated the majority of weight gain occurs in the first semester (approximately first 3 months of university) [6, 9, 87], some studies find that students weight continues to change beyond this point [6, 9, 88].

A second limitation is the sample size and the recruitment method. This study had 196 participants at baseline but previous studies have had larger samples (the average is 412 students when calculated across 17 studies (identified as most relevant to the current research) [6–11, 19, 35, 41, 42, 76, 87–91]. However, our analysis using effect sizes allowed us to identify associations that were meaningful given the current sample size. An additional issue for the sampling, which applies to any study involving participants having their weight measured, is a self-selection bias wherein those who are overweight may be more reluctant to participate [41, 88]. If participants expressed any concern regarding being weighed, they were told that they did not need to be informed of the number on the scale and that all information would be kept confidential. We are aware of only one participant who indicated that being weighed was the reason for not continuing with the study.

Further research is needed to address the limitations of the current study, especially regarding the problem encountered with the measure of alcohol consumption (see footnote above) and the modest follow up period. In addition, the current study did not incorporate a measure to assess drug intake or control circadian rhythmicity. Both drug use and circadian rhythmicity have been linked to impulsivity and weight gain [92, 93]. Explorations that extend into the later years of university would permit establishing whether critical study variables are associated with further weight/body fat change and changes in long term health behaviours in general. Research into the development of an intervention targeted at individuals who exhibit the cluster of traits identified as risk factors in the current study may also help to further explain the role they play in weight change during the first year at university.

## Conclusions

Entering university is a significant time of change and this can influence the development of (un) healthy habits. Previous research has typically used analyses which assume relationships between predictors and outcomes are homogenous across the sample. However, the current findings show this assumption may be misplaced. Examining distinct clusters of participants allowed for a more nuanced evaluation of the role of each variable in relation to weight gain. Even though there were distinct patterns of change for weight loss and weight gain across the four clusters, there were two unique patterns of change in psychological variables for those who were able to maintain their weight over the course of 3 months. This reinforces the idea that a one-sizes-fits-all approach may not be optimal for developing interventions to prevent the development of unhealthy habits and associated weight gain. Rather, different underlying psychological profiles can be associated with similar weight outcomes, and the relationship of a single variable with weight change may be impacted by other psychological factors.

## Data Availability

The dataset used in the analysis is available from the corresponding author upon request.

## Abbreviations

BMI: Body Mass Index
Kg: Kilograms
PA: Physical Activity
PACS: Physical appearance comparison scale
SD: Standard Deviation

## Footnotes

1 *Alcohol Consumption Habit:* Participants were also asked to complete a measure of habitual consumption of alcoholic beverages over the preceding 6 months. This measure was not included in the current analyses; it was not completed by 22% of participants at baseline and 16% of participants at time-2. It appeared that participants had skipped several questions and there was no reliable way of replacing the missing data.
